# Accuracy Assessment of a Wrist-Worn Oscillometric Blood Pressure Device Applied to the Upper Arm in Children

**DOI:** 10.7759/cureus.110605

**Published:** 2026-06-10

**Authors:** Akankshya Mahapatra, Mohd Saeed Siddiqui, Mohammad Haseeb, Avinash L Sangle, Vinod Ingale, Pradnya M Joshi, Prajakta Supe, Siddhant Sachdev, Rabiah Shaikh, Abinash Mohapatra

**Affiliations:** 1 Pediatrics, Mahatma Gandhi Mission Medical College and Hospital, Mahatma Gandhi Mission Institute of Health Sciences, Aurangabad, IND

**Keywords:** blood pressure (bp), british hypertension society, children, iso 81060-2, oscillometric, validation, wrist device

## Abstract

Background

Accurate blood pressure (BP) measurement in children is essential for detecting pediatric hypertension, yet validation evidence for wrist-worn oscillometric devices in this population is sparse. The nonstandard application of a wrist BP device to the upper arm, a clinically plausible scenario when cuff size is compatible with a child’s arm circumference, has not been previously evaluated. This study aimed to evaluate the accuracy and validation of a wrist-worn oscillometric BP device applied to the upper arm in children aged 3-8.5 years using brachial auscultatory mercury sphygmomanometry as the reference standard.

Methodology

This prospective, cross-sectional validation study enrolled 100 children (58 males; mean age = 4.9 ± 1.5 years) in a tertiary care hospital. A wrist-worn oscillometric device was applied to the upper arm and compared with brachial auscultatory mercury sphygmomanometry using the International Organization for Standardization (ISO) 81060-2:2018. A same-arm sequential protocol was used, yielding 300 paired comparisons. Accuracy was assessed using ISO Criteria 1 and 2, the British Hypertension Society (BHS) grading, and Bland-Altman analysis. Additionally, proportional bias regression and subgroup analyses were performed.

Results

The device met both ISO criteria for systolic BP (SBP; mean error = −4.62 ± 6.02 mmHg) and diastolic BP (DBP; −2.70 ± 5.19 mmHg). BHS grading was Grade A for DBP and Grade B for SBP. Bland-Altman 95% limits of agreement were −16.41 to +7.17 mmHg (SBP) and −12.88 to +7.48 mmHg (DBP). Significant proportional bias was observed for SBP (r = 0.370, p < 0.001) and DBP (r = 0.153, p = 0.008) with increasing underestimation at higher BP values. No sex differences were identified; a statistically significant but small age group difference was observed for DBP only.

Conclusions

This wrist-worn device applied to the upper arm met ISO validation requirements in 3-8.5-year-old children, and achieved BHS Grade A for DBP and Grade B for SBP. This device may be used as a screening tool in children of the tested age range, with confirmatory brachial auscultation recommended for elevated readings. The predominantly normotensive BP distribution in this cohort limits generalization of performance to hypertensive pediatric populations*.* Future studies should confirm our findings, recruit broader BP distributions, assess diverse anthropometrics, evaluate movement sensitivity, and explore pediatric-specific algorithms to reduce SBP proportional bias.

## Introduction

Accurate blood pressure (BP) measurement in children is fundamental to pediatric practice because elevated BP in childhood is frequently clinically silent, may indicate secondary pathology, and is an early determinant of future cardiovascular risk [1,2]. Even small measurement errors carry important consequences because pediatric BP classification relies on age-, sex-, and height-specific percentiles rather than fixed thresholds, making methodological rigor and validated devices essential [3-6].

Although upper-arm auscultation has traditionally served as the reference method for indirect BP assessment, clinical practice has shifted progressively toward automated oscillometric devices, driven in part by the phase-out of mercury sphygmomanometers and by the operational advantages of automation [6-9]. Oscillometric devices reduce observer dependence and are practical for routine and repeated measurements, but their accuracy cannot be assumed across devices, age groups, or measurement sites. For this reason, current validation science emphasizes independent device-specific assessment using standardized protocols, most notably the Association for the Advancement of Medical Instrumentation/European Society of Hypertension/International Organization for Standardization (AAMI/ESH/ISO) universal standard and ISO 81060-2:2018 [9,10].

Children pose particular validation challenges. Smaller limb dimensions, lower BP levels, higher heart rates, greater movement, and altered oscillometric waveform behavior reduce the transferability of adult validation data [11]. Devices accurate in adults may perform inadequately in children, necessitating dedicated pediatric validation [12]. Recent studies have focused on upper-arm devices, reinforcing brachial measurement as the preferred pediatric site [13,14].

Wrist devices introduce additional concerns. Oscillometric algorithms infer systolic and diastolic BP from the cuff-pressure oscillation envelope. Because mean arterial pressure is estimated most directly, systolic and diastolic values are more susceptible to algorithmic error when arterial properties vary [15-17]. Wrist measurement compounds these limitations through dependence on heart-level positioning and site-specific arterial differences. Accordingly, pediatric guidelines do not support wrist devices for diagnosing or managing hypertension when brachial measurement is feasible [5].

Evidence for wrist BP monitoring in children is sparse. In adolescents, a wrist monitor showed acceptable systolic but poorer diastolic agreement versus mercury sphygmomanometry [18]. To our knowledge, however, no published pediatric validation study has examined applying a wrist device to the upper arm in children, a clinically plausible off-label practice when cuff circumference matches a child’s arm. Because oscillometric algorithms are optimized for intended cuff geometry and site, such use cannot be presumed valid without formal testing.

This study aimed to evaluate the accuracy of a wrist-worn oscillometric BP device applied to the upper arm in children, using brachial auscultatory BP as the reference standard and internationally recognized validation criteria.

## Materials and methods

Study design and setting

This prospective, cross-sectional validation study was conducted in the outpatient department and pediatric ward of a tertiary care hospital from April 2024 to December 2025. Study procedures were based on the ISO 81060-2:2018 standard and corresponding practical recommendations for performing and reporting validation studies according to the AAMI/ESH/ISO Universal Standard [9,19,20]. Informed written consent was obtained from parents or legal guardians. Assent was obtained from children aged ≥7 years. Institutional Ethics Committee approval was obtained before enrolment (MGM-ECRHS/2024/120, dated 30 March, 2024). The Omron HEM-6161 device used in this study was a commercially purchased product. No funding, equipment provision, data access, or other support of any kind was received from Omron Healthcare or any affiliated entity. The manufacturer had no role in study design, data collection, statistical analysis, interpretation of results, or manuscript preparation.

Study population

Children aged 3-10 years attending pediatric outpatient and inpatient services were recruited by convenience sampling. A sample size of 85 is recommended by universal guidelines [19]. A total of 127 children were initially screened, of whom 100, with three paired readings each, were included in the final analysis. Both sexes were represented as stipulated by the validation protocol. Eligible participants were children aged 3-10 years with a mid-upper arm circumference of 13.5-20 cm, compatible with the cuff range of the test device, whose parents or guardians provided written informed consent. Children aged 3-10 years were initially screened; the final cohort comprised children aged 3-8.5 years, as defined by the arm circumference eligibility criterion. Children were excluded if any of the following were present: clinically significant cardiac arrhythmia; peripheral vascular disease or arteriovenous fistula in the study arm; acute febrile or hemodynamically unstable illness; arm circumference outside the usable cuff range of the test device; inability to obtain reliable auscultatory readings (e.g., Korotkoff sounds not clearly audible); and inability to remain seated and cooperative during the measurement sequence.

Devices

A calibrated mercury sphygmomanometer (Deluxe Mercurial BPMR120, manufactured by Industrial Electronic and Allied Products, Diamomd, India) was used as the reference standard, in accordance with the AAMI/ESH/ISO protocol. Auscultatory readings were obtained using a dual-head teaching stethoscope, enabling simultaneous listening by two trained observers. Appropriately sized pediatric cuffs were selected for each child such that bladder width was approximately 40% and bladder length covered 80-100% of the mid-upper arm circumference. The test instrument was a commercially available automated oscillometric wrist BP monitor (HEM-6161, manufactured by OMRON Healthcare, Vietnam) with a specified cuff circumference range of 13.5-21.5 cm. In this study, the device was applied in an off-label configuration to the right upper arm of participating children whose mid-upper arm circumference fell within the device’s stated cuff range. The cuff was positioned with its sensing region aligned over the brachial artery, and the arm was supported at heart level throughout the measurement procedure.

Validation team and measurement protocol

The validation team comprised one supervisor and two trained observers, consistent with the requirements of the Universal Standard. Both observers were trained in auscultatory BP measurement. The supervisor was responsible for operating the test device and overseeing protocol adherence and remained blinded to the reference readings. The two observers were blinded to each other’s readings and to the test device output. Both observers were trained pediatric clinicians experienced in auscultatory BP measurement. Before study initiation, a calibration session was conducted in which both observers independently measured BP in a set of pediatric patients under supervisor observation until inter-observer differences were consistently within 4 mmHg.

All measurements were performed in a quiet, temperature-controlled environment. The child was seated comfortably with their back supported, feet flat on the floor or appropriately supported, and the right arm resting on a flat surface at the level of the heart. A rest period of at least five minutes was observed before commencing measurements. Mid-upper arm circumference was measured at the midpoint between the acromion and olecranon processes using a non-stretchable tape and recorded in centimeters.

Following the ISO 81060-2:2018 same-arm sequential method, seven consecutive BP readings were obtained on the right arm in an alternating fashion between the reference and test devices, in the following fixed order: R1-T1-R2-T2-R3-T3-R4, where R denotes reference (auscultatory), and T denotes test-device measurements. A minimum 30-second inter-reading interval was observed between consecutive measurements to allow arterial recovery, consistent with ISO 81060-2:2018 requirements. For each reference measurement, both observers independently and simultaneously recorded systolic BP (Korotkoff phase I) and diastolic BP (Korotkoff phase V; phase IV if phase V was absent). Korotkoff phase V was the primary endpoint for diastolic BP (DBP) in all children; phase IV was used in approximately 9% of individual reference readings, occurring predominantly in children aged 3-5 years when phase V sounds were absent or indistinct. Phase IV readings were handled per protocol and were not analyzed separately given their small proportion. The mean of the two observers’ readings was used as the reference value. If the inter-observer difference exceeded 4 mmHg for either systolic BP (SBP) or DBP on any reference reading, that measurement was discarded and repeated. Three paired comparisons were derived per participant by matching each test device reading to the mean of the adjacent reference readings: T1 versus mean (R1, R2); T2 versus mean (R2, R3); and T3 versus mean (R3, R4). This yielded 300 paired comparisons across 100 participants.

Statistical analysis

Data were analyzed using Jamovi software version 2.4.1. Device accuracy was assessed against ISO 81060-2:2018 Criterion 1, requiring a mean difference of ≤±5 mmHg and standard deviation of ≤8 mmHg across all paired comparisons, and Criterion 2, requiring the standard deviation of subject-averaged differences to fall within protocol-specified thresholds conditional on the observed mean difference [20]. Agreement was additionally characterized using British Hypertension Society (BHS) grading based on cumulative proportions of absolute differences within ±5, ±10, and ±15 mmHg [21]. Bland-Altman analysis was performed to quantify mean bias and 95% limits of agreement [22]. To evaluate the agreement across the BP range, a proportional bias was assessed by regression of the Bland-Altman difference on the pairwise mean. Subgroup analyses were conducted by sex using independent-samples t-tests and by age group using one-way analysis of variance (ANOVA) with post hoc comparisons. A two-sided p-value of less than 0.05 was considered statistically significant. ISO 81060-2:2018 Criterion 1 was assessed across all 300 paired observations, and Criterion 2 was assessed using subject-averaged differences, in accordance with the protocol-specified analytical framework. This approach inherently accounts for within-subject structure and is the internationally mandated method for device validation. Secondary descriptive analyses (Bland-Altman, proportional bias regression, Pearson correlation) also used all 300 paired observations, consistent with standard reporting practice in ISO-based validation studies.

## Results

Study population

In total, 100 children (58 males (58%); 42 females (42%)) were enrolled, yielding 300 paired measurement sets comprising three consecutive device-reference reading pairs per participant. The mean age was 4.9 ± 1.5 years (range = 3-8.5 years). Mean height, weight, and body mass index were 102.2 ± 8.8 cm, 14.1 ± 2.4 kg, and 13.5 ± 1.6 kg/m², respectively. The mean upper-arm circumference was 14.4 ± 0.7 cm, consistent with the small-adult/pediatric cuff size used throughout the study. Baseline demographic and anthropometric characteristics are summarized in Table 1. Mean absolute inter-observer differences were 2.06 ± 1.38 mmHg (SBP) and 2.12 ± 1.50 mmHg (DBP); 99.7% of readings met the ≤4 mmHg criterion. Reference SBP ranged from 89 to 111.5 mm Hg and DBP from 54 to 70.5 mm Hg.

**Table 1:** Baseline demographic and anthropometric characteristics of study participants (N = 100). BMI: body mass index; MUAC: mid-upper arm circumference; Ref SBP: reference systolic blood pressure; Ref DBP: reference diastolic blood pressure

Characteristic	Value
Age (years), mean ± SD	4.9 ± 1.5
Height (cm), mean ± SD	102.2 ± 8.8
Weight (kg), mean ± SD	14.1 ± 2.4
BMI (kg/m²), mean ± SD	13.5 ± 1.6
MUAC (cm), mean ± SD	14.4 ± 0.7
Sex — Male, n (%)	58 (58)
Ref SBP (mm Hg), mean ± SD	99.5 ± 5.8
Ref DBP (mm Hg), mean ± SD	62.7 ± 4.5

ISO validation and BHS protocol grading

For Criterion 1, the mean device-reference difference was −4.62 ± 6.02 mmHg for SBP and −2.70 ± 5.19 mmHg for DBP, satisfying the required thresholds of ≤5 mmHg (mean) and ≤8 mmHg (SD) for both parameters. Notably, the mean SBP error approached the upper boundary of the permissible range, with a margin of only 0.38 mmHg. For Criterion 2, subject-level standard deviations were 3.42 mmHg for SBP and 3.34 mmHg for DBP, well within the interactive thresholds derived from the standard (SBP ≤ 5.16 mmHg; DBP ≤ 6.39 mmHg). The device thus satisfied all ISO 81060-2:2018 requirements in this pediatric cohort. For DBP, the device achieved BHS Grade A, with 69.7%, 90.3%, and 98.7% of readings falling within ±5, ±10, and ±15 mmHg of the reference, respectively. For SBP, the device achieved Grade B (54.0%, 84.7%, and 97.0% within the same thresholds), narrowly failing the Grade A requirements at the ±5 mmHg (54.0% versus ≥60%) and ±10 mmHg (84.7% versus ≥85%) boundaries (Table 2).

**Table 2:** ISO 81060-2:2018 Criteria 1, 2 and BHS protocol validation results (N = 300 measurement pairs). ISO 81060-2:2018 Criterion 1: mean error ≤ 5 mmHg and SD ≤ 8 mmHg; Criterion 2: threshold of individual-level SD differences: SBP ≤ 5.16, DBP < 6.39 mmHg [20]. BHS grading criteria: Grade A: ≥60%, ≥85%, ≥95% of readings within ±5, ±10, ±15 mmHg; Grade B: ≥50%, ≥75%, ≥90%; Grade C: ≥40%, ≥65%, ≥85%; Grade D: below Grade C. ISO: International Organization for Standardization; BHS: British Hypertension Society; SBP: systolic blood pressure; DBP: diastolic blood pressure; SD: standard deviation.

Criterion	Systolic BP	Diastolic BP
ISO 81060-2:2018 Criterion 1		
Mean error (threshold: ≤ 5 mmHg)	−4.62 mmHg (pass)	−2.70 mmHg (pass)
SD of differences (threshold: ≤ 8 mmHg)	6.02 mmHg (pass)	5.19 mmHg (pass)
ISO 81060-2:2018 Criterion 2		
Subject level SD of differences	3.42 (pass)	3.34 (PASS)
BHS protocol cumulative error distribution		
Within ±5 mmHg (Grade A: ≥60%)	54.00%	69.70%
Within ±10 mmHg (Grade A: ≥85%)	84.70%	90.30%
Within ±15 mmHg (Grade A: ≥95%)	97.00%	98.70%
BHS Grade	Grade B	Grade A

Bland-Altman agreement

**Figure 1:**
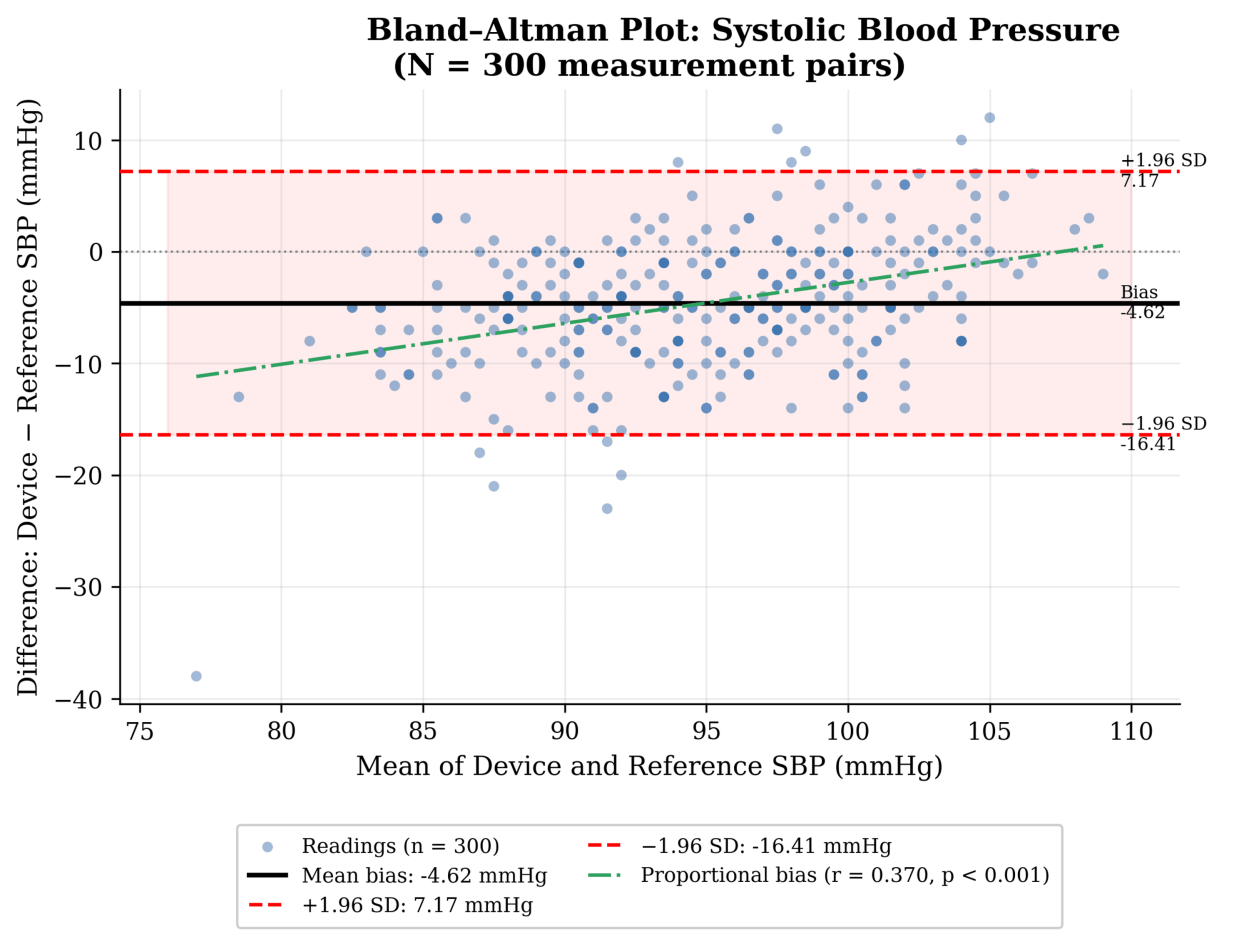
Bland-Altman plot of systolic blood pressure.

**Figure 2:**
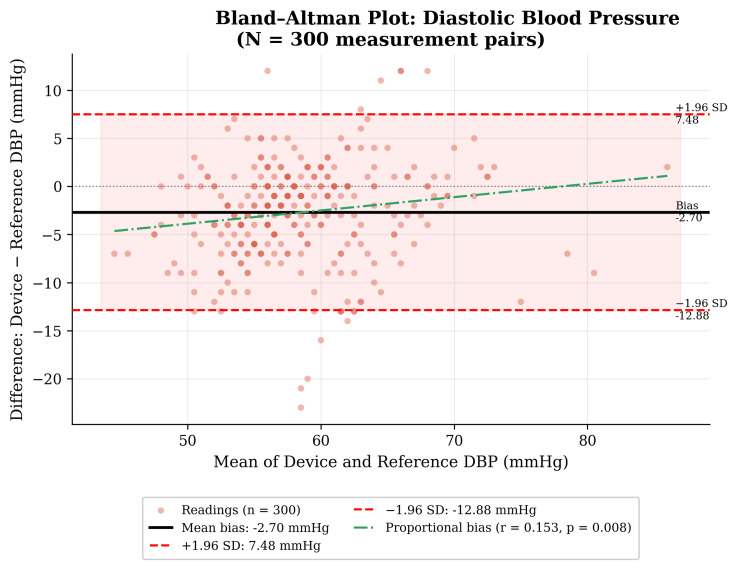
Bland-Altman plot of diastolic blood pressure.

The mean bias (device minus reference) was −4.62 mmHg (95% confidence interval (CI) = −5.30 to −3.93) for SBP and −2.70 mmHg (95% CI = −3.29 to −2.11) for DBP, both statistically significant (SBP: p < 0.001; DBP: p < 0.001), indicating systematic underestimation by the test device. The 95% limits of agreement were −16.41 mmHg (95% CI = −17.59 to −15.22) to +7.17 mmHg (95% CI = +5.99 to +8.36) for SBP and −12.88 mmHg (95% CI: −13.90 to −11.85) to +7.48 mmHg (95% CI: +6.46 to +8.51) for DBP. The SBP limits of agreement were notably wide, reflecting substantial inter-individual variability. Pearson correlation coefficients between device and reference readings were r = 0.635 (p < 0.001) for SBP and r = 0.664 (p < 0.001) for DBP. Bland-Altman plots for SBP and DBP are presented in Figure 1 and Figure 2, respectively, and agreement statistics are detailed in Table 3.

**Table 3:** Bland–Altman agreement statistics for systolic and diastolic blood pressure (N = 300 measurement pairs). Bias: device measurement minus reference (auscultatory mean of two trained observers). Pearson r was computed between device and reference readings. Proportional bias was assessed by regression of the difference on the pair-wise mean. SBP: systolic blood pressure; DBP: diastolic blood pressure; SD: standard deviation; CI: confidence interval; LOA: limits of agreement

Parameter	Systolic BP (value/statistic)	Systolic BP (p-value)	Diastolic BP (value/statistic)	Diastolic BP (p-value)
Mean bias ± SD (mmHg)	−4.62 ± 6.02	---	−2.70 ± 5.19	---
95% CI of mean bias	−5.30 to −3.93	---	−3.29 to −2.11	---
Lower LOA (95% CI)	−16.41 (−17.59 to −15.22)	---	−12.88 (−13.90 to −11.85)	---
Upper LOA (95% CI)	+7.17 (+5.99 to +8.36)	---	+7.48 (+6.46 to +8.51)	---
One-sample t-test (bias vs zero)	t = −13.29	<0.001	t = −8.99	<0.001
Pearson r (device vs. reference)	r = 0.635	<0.001	r = 0.664	<0.001
Proportional bias	r = 0.370	<0.001	r = 0.153	0.008

Proportional bias and subgroup analyses

To evaluate the agreement across the BP range, a proportional bias was assessed. Statistically significant proportional bias was identified for SBP (r = 0.370, p < 0.001) and DBP (r = 0.153, p = 0.008), with progressive underestimation at higher BP values. No sex differences were found for SBP (males = −4.32 ± 5.63 vs. females = −5.02 ± 6.51 mmHg; p = 0.319) or DBP (−2.53 ± 5.45 vs. −2.92 ± 4.84 mmHg; p = 0.526). SBP bias did not differ across age groups (p = 0.500). DBP bias differed significantly (F = 3.39, p = 0.036), with Games-Howell post hoc testing revealing a 1.58 mmHg difference between the 2-4 and 4-6-year groups (p = 0.033). All absolute biases remained within ISO limits.

## Discussion

This study evaluated the accuracy of a wrist-worn oscillometric BP device applied to the upper arm in children aged 3-8.5 years, using brachial auscultatory BP as the reference standard. Performance was assessed against ISO 81060-2:2018 requirements (Criterion 1 and Criterion 2) and summarized using BHS grading. Across 300 paired measurements from 100 children, the device satisfied both ISO criteria and achieved BHS Grade A for DBP and Grade B for SBP. However, SBP accuracy was borderline, with comparatively wide Bland-Altman limits of agreement and statistically significant proportional bias, findings that collectively support the device’s role as a screening instrument while underscoring the necessity of confirmatory brachial auscultation when wrist-derived SBP is elevated or borderline.

The rationale for evaluating this off-label configuration rests on the physical compatibility between the device’s cuff and the pediatric upper arm. The HEM-6161 cuff is specified for circumferences of 13.5-21.5 cm. In our cohort, the mean mid-upper arm circumference was 14.4 ± 0.7 cm, placing all participants within this operational range. Oscillometric BP measurement relies on detecting arterial wall oscillations transmitted through the compressed cuff. When the sensing marker is aligned over the brachial artery, as performed in this study, the fundamental detection mechanism is preserved regardless of application site. The relevant anatomical differences between the upper arm and wrist include limb geometry (cylindrical vs. tapered), tissue composition, and proximity of the sensor to the target artery. These differences are not trivially inconsequential, they may influence pressure transmission characteristics and oscillation envelope morphology, and constitute precisely the reason why independent empirical evaluation was necessary. The current study provides that empirical evidence, which cannot be extrapolated from the manufacturer’s wrist-application validation

The mean bias (test minus reference) was −4.62 ± 6.02 mmHg for SBP and −2.70 ± 5.19 mmHg for DBP, satisfying ISO 81060-2:2018 Criterion 1, which requires a mean difference of ≤5 mmHg with a standard deviation of ≤8 mmHg [3,4]. When contextualized within the pediatric validation literature, our DBP performance is consistent with multiple device evaluations demonstrating that DBP meets stringent agreement thresholds more reliably than SBP. Established pediatric validations, including the Omron 705 IT [23], the BpTRU [24], and the A&D UA-778 [25], have reported SBP and DBP differences broadly within the ≤5 ± ≤8 mmHg envelope using pediatric-appropriate protocols. Our SBP bias, although within ISO limits, trends closer to the upper permissible boundary than several upper-arm pediatric validations, a distinction of particular clinical significance given that pediatric BP classification relies on percentile-based thresholds where small differences in mmHg can alter diagnostic categorization.

The BHS grading results reinforce this asymmetry. For SBP, 54.0%, 84.7%, and 97.0% of readings fell within ±5, ±10, and ±15 mmHg, respectively, corresponding to Grade B; for DBP, 69.7%, 90.3%, and 98.7% met the same thresholds, yielding Grade A. This pattern, whereby oscillometric devices achieve higher BHS grades for DBP than for SBP, is well documented and mechanistically coherent. Oscillometric devices derive BP from the cuff-pressure oscillation envelope during deflation. The maximum oscillation amplitude corresponds most directly to mean arterial pressure, whereas SBP and DBP are inferred using proprietary or semi-empirical decision rules that are inherently more error-prone at the extremes of the envelope [15-17]. These sources of error are further amplified at the wrist, where accurate measurement depends critically on precise positioning at heart level and where peripheral waveform morphology and arterial compliance characteristics differ from those of the brachial artery [5].

Within-child variability of measurement error was low (SD = 3.42 mmHg for SBP; 3.34 mmHg for DBP), falling well below applicable ISO Criterion 2 thresholds [3,4]. This indicates reasonably consistent device performance across individual children under controlled conditions, even where population-level limits of agreement were wider. An important practical implication is that repeated measurements within the same child may attenuate random error but cannot eliminate systematic bias, a principle well established in validation science [22,26].

Bland-Altman analysis revealed 95% limits of agreement of -16.41 to +7.17 mmHg for SBP and -12.88 to +7.48 mmHg for DBP. Even if the mean bias between a test device and mercury auscultation is small, wide 95% limits of agreement imply that an individual child’s reading may differ substantially from the reference. This is clinically important because pediatric BP cut-points are percentile-based and can differ by only ~7-10 mmHg between key categories [4]. Outpatient pediatric studies comparing oscillometric devices with mercury have reported wide agreement limits/variability and demonstrated that such discrepancies can inflate hypertension classification rates [27,28]. This finding is consistent with pediatric studies demonstrating clinically important scatter despite acceptable mean differences, including arterial-line comparisons in critically ill neonates in which mean differences appeared acceptable, but prediction intervals were judged clinically unacceptably wide [29]. Similarly, in settings where invasive reference measurements are available, studies comparing noninvasive or wrist-based methods against arterial lines in children have demonstrated that substantial dispersion is common, reinforcing the need for caution when precision is required [30].

Statistically significant proportional bias was observed for both SBP and DBP, with underestimation increasing progressively as BP rose. This pattern is consistent with known limitations of oscillometric estimation algorithms, which are sensitive to pulse pressure, arterial compliance, and waveform morphology characteristics that may vary systematically at higher pressure levels [16,17]. From a clinical standpoint, proportional bias is most consequential precisely because it affects the children for whom accurate measurement matters most: those with readings near or above established decision thresholds. Contemporary pediatric guidance explicitly recommends that elevated oscillometric readings be confirmed by auscultation and acknowledges the limited evidence base for wrist devices in children [5].

For children aged 3-8.5 years, our findings support the use of this wrist-worn oscillometric device for routine screening, especially to identify clearly normal BP values. However, because SBP showed borderline accuracy and proportional bias, any elevated or borderline reading should be confirmed by brachial auscultation before classification or treatment decisions, consistent with pediatric guidance and with the broader evidence base cautioning against diagnostic reliance on wrist monitors in children. The clinical significance of the SBP limits of agreement (−16.41 to +7.17 mmHg) must be interpreted in the context of pediatric BP classification, which relies on narrow, age-, sex-, and height-specific percentile thresholds rather than fixed diagnostic cutoffs. As an illustrative example, for a five-year-old child of average height (approximately 110 cm), the 95th percentile SBP threshold is approximately 104 mmHg per AAP 2017 reference tables. Given the mean SBP underestimation of 4.62 mmHg and the proportional bias pattern, with underestimation increasing at higher BP values, a child with a true SBP at the 95th percentile could obtain a device reading 8-16 mmHg lower, potentially classifying them as normotensive. This represents a clinically meaningful false-negative screening risk. It must be emphasized that meeting ISO 81060-2:2018 criteria does not imply clinical interchangeability with auscultatory measurement; ISO thresholds were established as device performance benchmarks and do not account for the narrow classification margins inherent to percentile-based pediatric BP interpretation. Confirmatory brachial auscultation for any borderline or elevated reading is therefore not merely advisable but necessary

Strengths include the application of ISO and BHS frameworks with comprehensive analyses reported per Universal Standard expectations. Limitations include single-center design, single-device testing, under-representation of elevated BP ranges constraining inference where proportional bias is most relevant, and absence of testing under real-world pediatric conditions, such as movement and agitation. Future studies should recruit broader BP distributions (including more elevated/hypertensive-range readings), test performance across diverse anthropometrics, explicitly assess movement sensitivity, and evaluate whether pediatric-specific wrist algorithms can reduce SBP proportional bias.

## Conclusions

This wrist-worn oscillometric BP device applied to the upper arm met ISO 81060-2:2018 validation requirements in children aged 3-8.5 years and achieved BHS Grade A for DBP and Grade B for SBP. This device may be used as a screening tool in children of the tested age range, but confirmatory brachial auscultation is necessary when readings are elevated or near decision thresholds. Passing ISO 81060-2:2018 criteria should not be interpreted as equivalence with auscultatory measurement for clinical decision-making. ISO thresholds define device performance benchmarks and do not account for the narrow margins of paediatric percentile-based BP classification. The wide SBP limits of agreement (−16.41 to +7.17 mmHg) carry a real risk of clinically significant misclassification in individual children, particularly at BP values near established percentile thresholds. The statistically significant proportional bias, with progressive SBP underestimation at higher BP levels, is especially concerning for a screening application, as it is precisely in children with elevated true BP that device accuracy is most consequential. Future studies should confirm our findings, recruit broader BP distributions, assess diverse anthropometrics, evaluate movement sensitivity, and explore pediatric-specific algorithms to reduce SBP proportional bias.
